# Prophylactic Use of Meloxicam and Paracetamol in Peripartal Sows Suffering From Postpartum Dysgalactia Syndrome

**DOI:** 10.3389/fvets.2020.603719

**Published:** 2020-12-08

**Authors:** Alexandra Schoos, Ilias Chantziaras, Jordy Vandenabeele, Evelien Biebaut, Evelyne Meyer, An Cools, Mathias Devreese, Dominiek Maes

**Affiliations:** ^1^Unit of Porcine Health Management, Department of Reproduction, Obstetrics and Herd Health, Faculty of Veterinary Medicine, Ghent University, Merelbeke, Belgium; ^2^Unit of Social Sciences, Fisheries and Food, Research Institute for Agriculture, Melle, Belgium; ^3^Department of Pharmacology, Toxicology and Biochemistry, Faculty of Veterinary Medicine, Ghent University, Merelbeke, Belgium; ^4^Department of Nutrition, Genetics and Ethology, Faculty of Veterinary Medicine, Ghent University, Merelbeke, Belgium

**Keywords:** NSAID, acetaminophen, sow, inflammation, fever, PPDS, prophylaxis, piglet

## Abstract

Postpartum dysgalactia syndrome (PPDS) is a major economic problem in modern sow farms. General treatment of PPDS consists of the use of oxytocin to promote milk ejection and non-steroidal anti-inflammatory drugs (NSAIDs) to alleviate inflammatory processes. So far, studies investigated the use of a single administration of NSAIDs after parturition in healthy and non-healthy sows. The current study investigated whether administration of meloxicam or paracetamol in sows prior to parturition improves sow and piglet health as well as performance in a farm with PPDS problems in sows. Sixty sows and 978 piglets from a Belgian farrow-to-finish farm were enrolled. Sows were randomly divided into three groups: a non-treated control group, a meloxicam-treated group and a paracetamol-treated group. Treatment was administered orally for 7 days from gestation day 113 onwards. Performance and health parameters investigated in sows were gestation length, farrowing duration, litter characteristics, colostrum yield and quality (Immunoglobulin G), litter weight gain, weaning-to-estrus interval, pregnancy rate, rectal temperature, acute phase proteins and inflammatory markers serum amyloid A, haptoglobin, interferon γ, interleukin 1β and 6 backfat, constipation and feed refusal. Performance and health parameters in piglets were birthweight, average daily weight gain, colostrum intake and mortality. Paracetamol-treated sows showed a significantly (*P* = 0.04) lower rectal temperature (mean ± SD: 38.09 ± 0.18°C) than the meloxicam-treated sows (38.24 ± 0.18°C), but not than the control group (38.22 ± 0.18°C). Sows of the paracetamol-treated group had a significantly (*P* = 0.001) longer gestation length (116.3 ± 0.9 days) than sows of the control group (115.3 ± 0.6 days), but not than meloxicam-treated sows (115.9 ± 0.9 days). No significant differences between the three groups were found for all the other parameters. In conclusion, the prophylactic oral administration of either meloxicam or paracetamol for 7 days starting 2 days prior to farrowing did not show beneficial effects on both health and performance parameters of sows and piglets.

## Introduction

The peripartum period is critical in the reproductive cycle of sows. One of the major problems in modern farms is the postpartum dysgalactia syndrome (PPDS). Sows have reduced colostrum and milk production but often show little or no obvious inflammation with a variable clinical picture (1). Piglets mainly show growth retardation due to insufficient colostrum and milk intake (2). The economic impact of PPDS can be substantial (3), including increased piglet mortality, diarrhea, higher treatment costs and a lower weaning weight. It also leads to increased replacement rates of the sow population and more labor for care and treatment.

Based on a questionnaire-based study in Flanders (Belgium), 34% (37/110) of the farms encounter PPDS problems (4). The main risk factors for PPDS are farrowing induction (30%), *ad libitum* feeding shortly after farrowing (26%), moving sows < 4 days before expected farrowing from the gestation to the farrowing unit (34%) and no farrowing supervision (20%). Genetic predisposition might be a risk factor for PPDS as well (5). A German study investigated three farms over a 2-year period and observed a PPDS prevalence ranging from 1.8 to 20.8%, with seasonal fluctuations (6). A recent Swiss study showed a PPDS prevalence in herds up to over 75%, whereas the overall average prevalence was around 37% (7). Nevertheless, it is difficult to compare such prevalence and incidence data, as criteria defining PPDS-related clinical signs vary between studies (8). Indeed, the pathophysiology of PPDS is complex and not yet fully elucidated. The condition starts already before farrowing, and in general, the interaction of three major factors is considered important: stress, the combination of feed composition and feeding strategy, and endotoxemia caused by an infectious agent (9, 10).

Treatment consists of the use of oxytocin to promote milk ejection and non-steroidal anti-inflammatory drugs (NSAIDs) to alleviate inflammatory processes. It was recently shown that the most effective control measures to fight PPDS were the use of a transition feed with expandable raw fiber prepartum to avoid constipation, a good PPDS diagnostic (i.e., reddening of the teats, apathy and lack of appetite, constipation, hypogalactia, vaginal discharge) and the treatment with NSAIDs and oxytocin in case of such clinical signs (7).

NSAIDs have an anti-inflammatory, analgesic and antipyretic action by inhibiting the cyclooxygenase (COX) activity and thus the prostaglandin synthesis. In the present study, we investigated the prophylactic use of meloxicam and paracetamol in sows in a farm with a PPDS history. Meloxicam is a COX-2 selective NSAID in pigs and acts exclusively peripherally (11). In contrast, paracetamol is a NSAID-like drug with central antipyretic and analgesic action and without peripheral anti-inflammatory action (12). Due to its strong antipyretic action, it has been investigated mainly for treating respiratory disease in fatteners (13, 14). Up to now, no studies have investigated its potential effect in problems of periparturient sows. According to the leaflet, meloxicam is indicated for animals showing puerperal septicemia and toxemia, and paracetamol is indicated for animals having fever (Supplementary Files 1, 2). Meloxicam is indicated to be administered once, or if needed twice, with an interval of 24 h. Paracetamol is indicated to be given over 5 days. Both products are registered to be administered to sows during gestation and lactation.

The effects of prophylactic administration of NSAIDs before parturition and lasting until the 1st days postpartum to sows in a farm encountering PPDS have not yet been investigated. Nevertheless, such preventive use might be important to prevent the cascade of inflammatory processes leading to PPDS. Therefore, the aim of this study was to investigate if prophylactic oral administration of meloxicam or paracetamol to periparturient sows can improve sow and piglet health and performance in a farm encountering PPDS in sows.

## Materials and Methods

### Farm Description and Study Population

The experimental study protocol was approved by the Ethical Committee of the Faculty of Veterinary Medicine and the Faculty of Bioscience Engineering, Ghent University (EC2019-26), as well as by the Flemish governmental agency for animal welfare (DWZ/ER/20/1.15/). All interventions and recordings in this study were performed by veterinarians or by senior undergraduate veterinary medicine students under supervision of the first author. The study was performed in a Belgian farrow-to-finish farm with a history of PPDS: low feed intake and constipation around farrowing, long farrowing duration (> 6 h), dysgalactia and high preweaning mortality (on average 18.4% in the period between November 2018 and November 2019). According to the farmer's records, sows had a gestation length of 116 days. The farm had in total around 500 Danbred sows (Landrace x Yorkshire), worked in a 4-week-batch production system weaning at 21 days. The sows were inseminated with semen from German Piétrain boars. The farrowing unit consisted of one compartment with 100 conventional farrowing crates conform to the Council Directive 2001/88/EC laying down minimum standards for the protection of pigs. The sows and gilts were transferred to the farrowing unit three to 7 days before the expected farrowing date.

Until the day of entrance to the farrowing unit, sows were fed with gestation feed followed by a transition feed until 2 to 3 days after the last sow had farrowed. From then onwards until weaning, sows were fed with lactation feed. Precise composition of the different feeds can be found in Supplementary Table 1. The drinking water came from a deep well (76 m) and peroxides were added. The sows had *ad libitum* water via a drinking nipple system and they received extra water around farrowing with a trowel if they were not eating well. The water quality parameters (Inagro, Rumbeke-Beitem, Belgium) were within the reference values with a pH of 7.78 and a total hardness of 3.34 F°. The ventilation consisted of a channel ventilation system and the ambient temperature in the farrowing unit varied between 24.5 and 25°C. Gilts were vaccinated against *Mycoplasma hyopneumoniae*, porcine circovirus 2 (PCV2), porcine reproductive and respiratory syndrome virus (PRRSV), parvovirus and *Erysipelothrix rhusiopathiae*. Sows were vaccinated against *Bordetella bronchiseptica* and *Pasteurella multocida* toxin type D, *Escherichia coli, Clostridium perfringens* type C and *Clostridium novyi* type B, *Glaesserella parasuis*, parvovirus and PRRSV. Piglets were vaccinated 2 days before weaning against *Mycoplasma hyopneumoniae* and PCV2.

Using a randomized complete block design based on parity, all sows and gilts of one batch, i.e., 78 sows and 18 gilts, were considered for the study and were assigned to either one of three treatment groups: non-treated control group (CG), meloxicam-treated group (MG) or paracetamol-treated group (PG). Animals from the different groups were moved in the farrowing crates based on sequence of entering to the farrowing stable irrespective of treatment group. No sow suffered from shoulder ulcers or severe lameness. The first 16 sows and the first four gilts that farrowed of each treatment group were enrolled in the study. This resulted in 20 animals (sows, gilts) per treatment group (*n* = 60) and the offspring. Remaining sows and gilts were not further considered and all collected data until then (rectal temperature, defecation, feed intake and backfat) was withdrawn. Sows and gilts that farrowed prior to gestation day 115 and after day 117 were not anymore considered for the study, as according to the protocol, animals had to be medicated for at least 2 days before farrowing and at least 2 days after farrowing, with in total seven treatment days. Induction of farrowing with prostaglandin F2α (PGF2α) was not permitted, as this could influence the effect of meloxicam and paracetamol. Piglets remained with their sow for 24 h postpartum. Thereafter, cross-fostering and split suckling were allowed within the same treatment group.

### Medication

**Figure 1:**
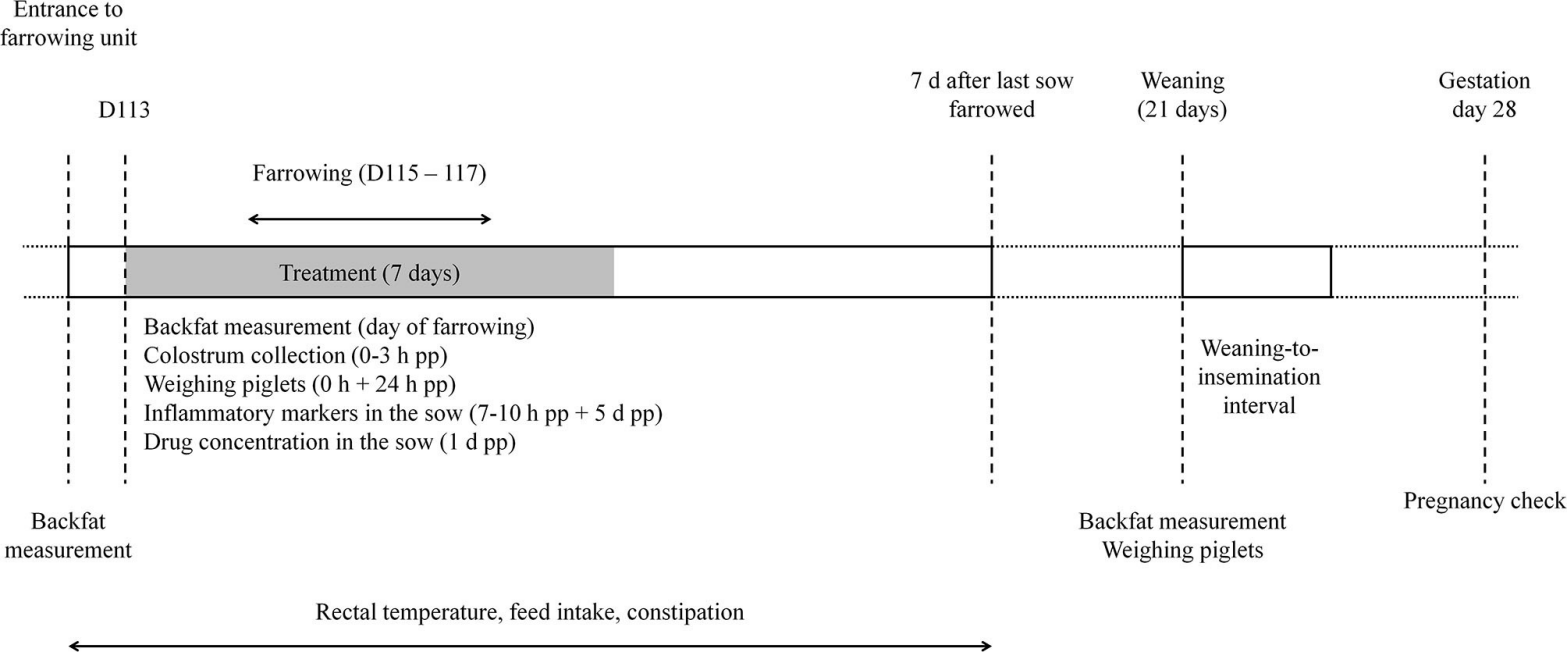
Timeline of the study with specific interventions. D, day of gestation; d, days; h, hours; pp, postpartum.

Figure 1 gives an overview of the study setup. Animals were treated once daily for 7 days from gestation day 113 onwards until at least 2 days after farrowing (Figure 1). Animals from the treatment groups received orally 0.4 mg/kg bodyweight (BW) meloxicam (Metacam 15 mg/ml oral suspension, Boehringer Ingelheim) or 30 mg/kg BW paracetamol (Pracetam 400 mg/ml, Ceva). Both doses corresponded to the European label dose. The bodyweights of the sows and gilts were estimated based on the farm's latest slaughterhouse results, namely 280 kg for sows and 250 kg for gilts. Medication was administered each day by the same person per treatment group with a syringe directly in the mouth of the animals just before the first feeding in the morning. The non-treated control animals did not receive any treatment.

### Sample Collection, Measurements and Calculations of Parameters

Backfat thickness of sows was measured left and right at the P2 position (65 mm off the mid line at the level of the last rib; Renco Lean-Meater, MN, USA) at the moment of entrance to the farrowing unit, at farrowing and at weaning (15). Rectal temperature, feed intake and defecation were monitored daily between 9 and 11 A.M. by the same person, who was blinded to treatment. These parameters were recorded from the day of entering the farrowing unit until 7 days after the last sow had farrowed (Figure 1). Feed intake was not quantified. Either the trough was empty (no feed leftover) or not (feed leftover). Mild constipation was defined as one or two consecutive days without defecation. If sows didn't defecate for three or four days, it was considered as severe constipation, and if sows didn't defecate for five or more days, it was considered as very severe constipation, i.e., obstipation (16). To indicate whether a sow was clinically PPDS-affected or not, retrospective evaluation was performed. More specifically, at least two of the three indicators had to be present: rectal temperature ≥ 39.5°C, feed leftover and obstipation (defined as very severe constipation).

The parturitions were supervised for 24 h a day and litter characteristics (total born, live born, dead born and mummified piglets) were recorded until all 60 sows and gilts had completed farrowing. Colostrum (25–40 ml) was collected from different teats (pectoral, abdominal and inguinal) within 0–3 h after onset of parturition. Immediately after birth, piglets were weighed (BWB) and received an individual eartag. Twenty-four hours (23–25 h) after birth of the piglet, they were weighed again (BW24) in order to estimate colostrum intake (CI) and colostrum yield (CY). Colostrum intake was estimated based on the mechanistic model as described by Theil et al. (17), which includes BWB (kg), weight gain within the first 24 h after birth (WG; g) and duration of CI (D; minutes). The equation is as follows: CI = −106 + 2.26 ^*^ WG + 200 ^*^ BWB + 0.111 ^*^ D – 1.414 WG/D + 0.0182 ^*^ WG/BWB. In case negative values were obtained, CI was considered to be zero (18, 19). Estimated CY was calculated as the sum of the individual piglet's CI within a litter. Potential CI of piglets dying within 24 h after birth was not considered. At weaning, piglets were weighed again (BWW) in order to determine the ADWG and the average daily weight gain per litter. Mortality of piglets was recorded for the whole lactation period. After weaning, the weaning to insemination interval of each sow was recorded. Four weeks after insemination, pregnancy testing was done by transabdominal ultrasound and pregnancy rate was calculated for each group.

For inflammatory marker analyses, sow blood samples were taken at two time points, namely between 7 and 10 h and 5 days after farrowing. For pharmacological analyses, the blood sample was taken the day after farrowing at the presumed time point of maximal drug concentration (T_max_), namely 2.5 h and 30 min after oral administration for meloxicam and paracetamol, respectively (20, 21). For serum, blood samples were centrifuged at 1,000 *g* for 10 min at 4°C, aliquoted à 500 μL and stored at −80°C until analysis. For plasma, blood samples were centrifuged at 5,250 *g* for 5 min at 4°C, aliquoted à 1,500 μL and stored at −20°C until analysis.

### Analytical Methods

#### Quantification of Meloxicam and Paracetamol in Plasma

Plasma concentrations of meloxicam and paracetamol were determined using an in-house validated high-performance liquid chromatography (HPLC)-UV method previously described by Baert and De Backer (meloxicam) and Neirinckx et al. (paracetamol) (21, 22). Samples were prepared by pipetting 500 μL plasma into screw-capped Pyrex tubes. Each sample was spiked with 25 μL of the internal standard working solution of 100 μg/mL phenacetine (paracetamol) or piroxicam (meloxicam). After spiking and vortexing briefly, 100 μL HCl 1 N were added. Five milliliters of tert-butyl methyl ether (paracetamol) or diethylether (meloxicam) were then added, and the samples were allowed to extract for 20 min by gentle rolling. After centrifugation (4,000 rpm for 10 min at 4°C) the upper layer (4.5 mL) was transferred into a clean screw-capped Pyrex tubes and evaporated under nitrogen at 40°C. The residue was redissolved in 200 μL of mobile phase A, vortexed for 15 s and transferred into vials for HPLC and 10 μL were injected onto the LC-UV. The stationary phase consisted of an UHPLC Acquite BEH C18 column (1.7 μm, 2.1 × 50 mm; Waters, Zellik, Belgium). The mobile phase consisted of 0.01 M glacial acetic acid in water (A) and acetonitrile (B) and a gradient elution program was used. Detection was done at λ = 355 nm (meloxicam) and λ = 248 nm (paracetamol). The limit of quantification (LOQ) was set at 0.1 μg/mL (paracetamol) or 0.05 μg/mL (meloxicam) with a linear range up to 5 μg/mL. Concentrations out of the upper limit of the calibration curve were re-analyzed after appropriate dilution with blank plasma.

#### Acute Phase Proteins and Pro-inflammatory Cytokines Analysis

Analyses of the important porcine acute phase proteins (APPs) serum amyloid A (SAA) and haptoglobin (Hp) were performed at the veterinary laboratory Zoolyx (Aalst, Belgium). Serum samples were analyzed by an immunoturbidimetric assay (SAA: Eiken Chemical, Japan; Hp: Tridelta, Ireland), which was performed according to manufacturer's instructions on an automated chemistry platform (Cobas 8000, Roche Diagnostics, Switzerland). Pro-inflammatory cytokines interferon γ (IFN-γ), interleukin 1β (IL-1β) and interleukin 6 (IL-6) were analyzed in serum with a multiplex immunoassay (Custom Porcine ProcartaPlex Multiplex Immunoassay, ThermoFisher Scientific, USA) according to manufacturer's instructions. For data analyses, the lower limit of detection (LOD) was provided by the manufacturer (1.25 ng/L, 0.09 ng/L and 0.43 ng/L for IFN-γ IL-1β, and IL-6, respectively), while the LOQ was the lowest concentration of the standard curve fitting still in the recovery range of 70–130%. Serum concentrations ≤ LOD were converted to the mean between 0 and LOD. Concentrations ≥ LOD, but ≤ LOQ, were converted to the mean between LOD and LOQ.

### Colostrum Analysis

For measurement of porcine IgG in colostrum, a commercially available ELISA (Pig IgG ELISA Core Kit, Pink-ONE, Komabiotech, Seoul, Korea) was used according to manufacturer's instructions. Colostrum samples were diluted 1:500,000 and 1:1,000,000. The intra-assay coefficient of variation was 2.4% ± 1.7 (mean ± SD). Colostrum samples were analyzed on three ELISA plates with an inter-assay variation of 4.3% (1.5–9.2%) based on two reference colostrum samples. All samples were analyzed in duplicates and calculation of the IgG value based on calibration curves from eight standard concentrations was performed using the DeltaSOFT program (BioMetalics Inc., Princeton, USA).

### Statistical Analyses

For the power calculations of this study a two-means two-sided equality test was used (23). The rectal temperature of sows was considered as primary efficacy criterion in this study with α = 0.025, a power of 80%, means of 38.0°C and 38.4°C and a SD of ± 0.4°C. The average daily weight gain per litter per sow was determined as primary efficacy criterion for performance of piglets. Therefore, an α = 0.025, a power of 80%, means of 2.68 kg and 2.55 kg and a SD of ± 0.130 kg were chosen. Those two parameters were chosen to calculate the sample size. A sample size of 19 sows per treatment group resulted from this power calculation.

Descriptive information about the various parameters included in this study was calculated. The assumptions of normality and homogeneity of variance were tested by examining histograms, normal probability plots of residuals, and plots of studentized residuals vs. predicted values. Statistical analyses were performed using IBM SPSS version 26® (Armonk, New York, USA). Confounding effects were checked during modeling by evaluating changes in parameter estimates. Any confounders were retained upon significance (*P* ≤ 0.05). When needed, dependent variables were transformed accordingly to meet the assumptions of the statistical model used (e.g., in the case of farrowing duration, a natural log transformation was used to transform the positively-skewed dependent variable). Within each dependent variable, Bonferroni correction for multiple categories (CG, MG, PG) was applied, but no correction was further applied for multiple testing (i.e., all the different tests performed), as this was deemed a too strict criterion in relation to the needs of this study.

For gestation length, farrowing duration, litter characteristics, CY, CI, IgG concentration in colostrum, daily litter weight gain, backfat loss, ADWG, weaning-to-insemination interval, acute phase protein and cytokine concentrations, ANOVA or Kruskal-Wallis tests were applied to signify any differences between the treatment groups. The means of the piglet mortality rates were compared between the treatment groups. Prior comparisons, the 1st day mortality rates were transformed (arcsine square root). Finally, a Kaplan-Meier survival analysis was conducted with pairwise log rank comparisons. Chi-square tests were used to compare the pregnancy rates between the treatment groups. Comparison of rectal temperature between treatment groups was performed by a linear mixed model. Each sow was listed as “subject,” and the sampling as “repeat.” An autoregressive covariance matrix of the first order was used for the repeated covariance structure. Rectal temperature was recorded prior (pre-treatment period), during (treatment period) and after treatment (post-treatment period). To correct for this, treatment period was included as a fixed factor in the model. For the presence of feed leftover or not, generalized estimating equations (GEE) were used and a repeated binomial logistic model with treatment groups as fixed effect and sampling days as repeated was performed. An autoregressive first order correlation matrix for repeated measurements was set. To correct for overdispersion, we rescaled the covariance matrix based on the Pearson Chi square statistic. Per period (pre-treatment, treatment and post-treatment period), the averages of the fecal score values were compared with the use of a Chi square test. To include all scoring instances and acknowledge the ordinal nature of the fecal score, we fitted a repeated multinomial cumulative logit model with treatment groups as fixed effect and the three scoring moments as repeated. An autoregressive first order correlation matrix for repeated measurements was again set.

## Results

### Plasma Concentrations of Meloxicam and Paracetamol

**Figure 2:**
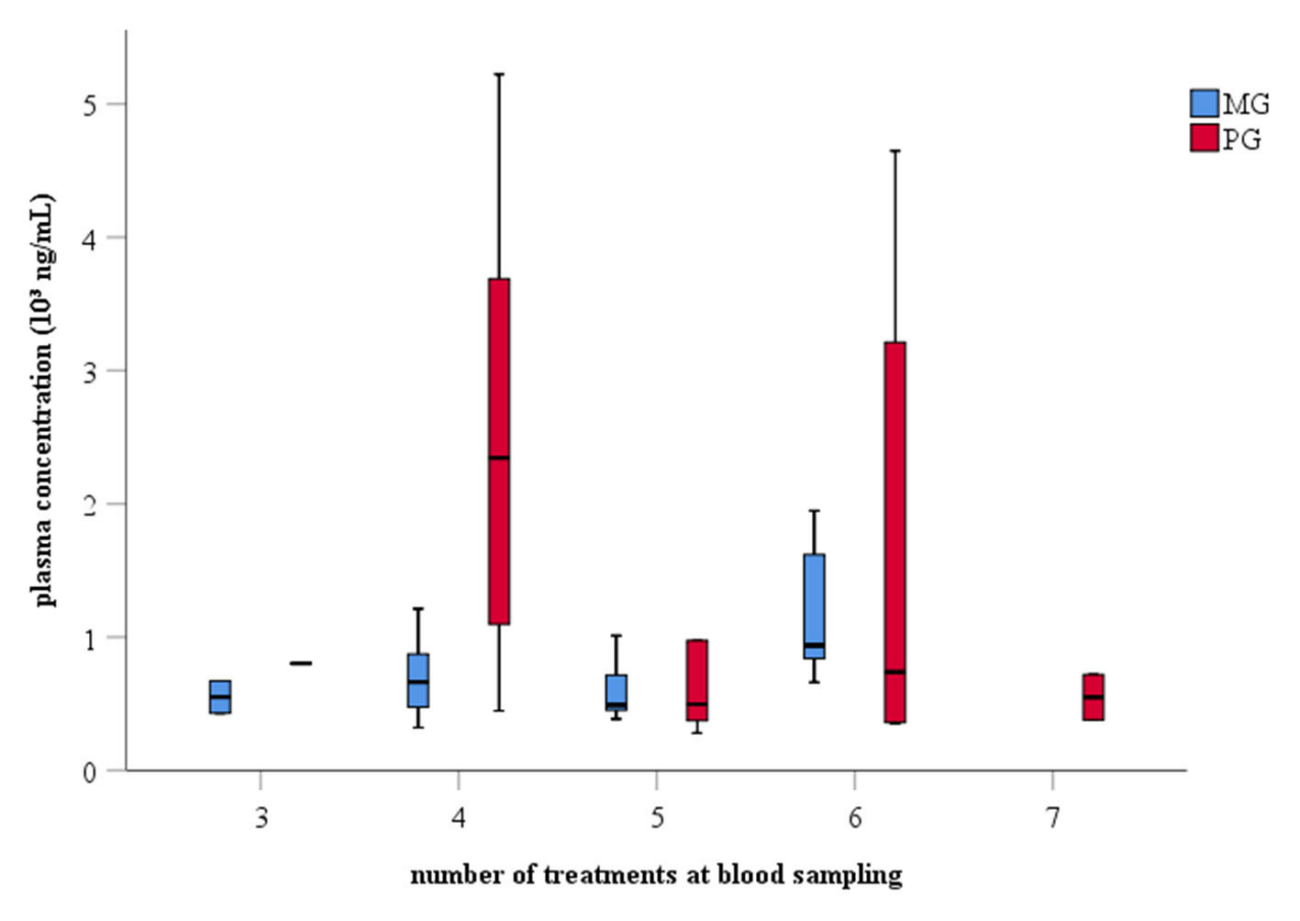
Boxplot of plasma drug concentration and number of treatments at blood collection from sows treated with meloxicam (0.4 mg/kg BW, *n* = 20) or paracetamol (30 mg/kg BW, *n* = 20). Blood samples were collected at expected T_max_ of the respective drug, i.e., 30 min and 2.5 h post-treatment for paracetamol and meloxicam, respectively, 1 day after farrowing. BW, bodyweight; T_max_, time point of maximal drug concentration in plasma.

The average (± SD) number of treatments at the moment of blood sampling was 5 (± 1) days in both groups with ranges from 3-6 to 3-7 days in MG and PG, respectively (Figure 2). Meloxicam-treated sows had a mean (± SD) plasma concentration of 786 (± 416) ng/mL with a range from 320 to 1,949 ng/mL. Paracetamol-treated sows had a mean (± SD) plasma concentration of 3,548 (± 9,126) ng/mL with a range from 281 to 41,738 ng/mL (Supplementary Table 2).

### Performance Parameters in Sows

#### Gestation Length, Farrowing Duration, and Litter Characteristics

The mean gestation lengths of the sows from the different groups are indicated in Table 1. The gestation length of the PG was significantly longer than the one of the CG (*P* < 0.001), but not than that of the MG (*P* = 0.4). Farrowing lasted on average 411 min. Differences between groups were not significant. The number of total born piglets was almost equal in all three groups. The number of piglets born alive, dead or as a mummy were not significantly different between the three groups with overall averages of 16.4 piglets, 3.3 piglets and 0.9 mummified piglets, respectively (Table 1).

**Table 1:** Effects of meloxicam (0.4 mg/kg BW) and paracetamol (30 mg/kg BW) on sow's performance.

	**Control (*****n*** **= 20)**		**Meloxicam (*****n*** **= 20)**		**Paracetamol (*****n*** **= 20)**		
	**Mean**	**SD**	**Mean**	**SD**	**Mean**	**SD**	***P*-value (test used)**
Parity	3.4	1.9	3.1	2.6	3.6	2.1	0.5 (K-W)
Gestation length (d)	115.3^a^	0.6	115.9^ab^	0.9	116.3^b^	0.9	0.002 (K-W)
Farrowing duration (min)	359	170	453	258	422	279	0.5^†^ (ANOVA)
Litter characteristics							
Total born	20.9	5.0	21.0	4.3	20.0	4.3	0.8 (K-W)
Live born	16.7	5.1	16.2	3.6	16.3	4.3	0.8 (K-W)
Dead born	3.1	3.5	3.7	2.8	3.1	2.9	0.6 (K-W)
Mummified piglets	1.1	1.5	1.1	1.9	0.6	1.0	0.4 (K-W)
Colostrum yield (kg)	4.70	1.63	4.46	1.08	4.51	1.18	0.8 (ANOVA)
Colostrum IgG (g/l)	120.4	48.6	117.2	41.2	109.8	33.7	0.7 (ANOVA)
Litter weight gain (kg/day)	2.14	0.49	2.21	0.39	2.06	0.52	0.6 (ANOVA)

*Sows were treated for 7 days from gestation day 113 onwards*.

a−b *Values in the same row not sharing the same superscript are significantly different at P < 0.05 in either the Kruskal-Wallis test (K-W) or the test the means for equality (ANOVA). Tests are adjusted for all pairwise comparisons within a row using the Bonferroni correction*.

† *Parameter transformed (natural logarithm) to test the means for equality*.

#### Colostrum Yield and IgG Concentration, Daily Litter Weight Gain and Piglet Mortality

The average CY, IgG concentration and daily litter weight gain from the different groups are indicated in Table 1. Piglet mortality during the 1st week was the highest in litters of non-treated sows, followed by the mortality of piglets of the PG and the MG (Table 2). The overall preweaning mortality was 23.4%. All differences of above mentioned parameters were not significant.

**Table 2:** Piglet mortality in 332 piglets of 20 non-treated sows, 321 piglets of 20 meloxicam-treated sows (0.4 mg/kg BW) and 325 piglets of 20 paracetamol-treated sows (*n* = 20) (30 mg/kg BW).

	**Control**	**Meloxicam**	**Paracetamol**	
	**%**	**%**	**%**	***P*-value**
Mortality 24 h (%)	5.4	4.7	6.5	0.9^†^
Mortality 1st week (%)	21.7	17.8	19.1	0.2
Preweaning mortality (%)	25.3	22.4	22.5	0.6

*Sows were treated for 7 days from gestation day 113 onwards*.

† *Parameter transformed (arcsine square root) to test the means for equality*.

#### Post-Weaning Performance

One sow belonging to the MG and four sows belonging to the PG were culled after weaning and transferred to the slaughterhouse. No post-mortem examinations were performed at the slaughterhouse. In two sows (one of the MG and one of the CG) no estrus was detected. The weaning to insemination interval was comparable in all groups averaging 4 days (3.7, 3.8, 3.9 days in CG, MG, and PG, respectively), with a total range of 1–5 days. The pregnancy rate was 89% (17/19 sows), 94% (17/18 sows) and 100% (16/16 sows) in the CG, MG and PG, respectively, and did not differ significantly between groups.

### Health Parameters in Sows

#### Clinical Signs of PPDS and Risk Factors

Sows of the CG and MG had an average (± SD) rectal temperature of 38.2 ± 0.3°C and 38.2 ± 0.3°C, respectively. Pairwise comparisons of the treatment groups showed that sows of the PG had a statistically significant lower rectal temperature (38.1 ± 0.3°C) compared to sows of the MG (*P* = 0.04), but not the CG (*P* = 0.09). Over the whole recording period, five sows of each group had at least once a rectal temperature ≥ 39.5°C. Rectal temperatures for all groups ranged from 35.4 to 39.6°C, from 36.5 to 40.5°C and from 37.0 to 40.6°C in the pre-treatment, treatment and post-treatment period, respectively. Average rectal temperatures in these three periods were 37.8 ± 0.3°C, 38.2 ± 0.2°C, and 38.4 ± 0.2°C, respectively. The mixed model results indicated statistically significant differences between the treatment groups (*P* = 0.03) and the treatment period (*P* < 0.001). Neither vaginal discharge, shoulder ulcers, edematous udders or other clinical signs, nor additional antibiotic treatment were noticed during this study.

**Figure 3:**
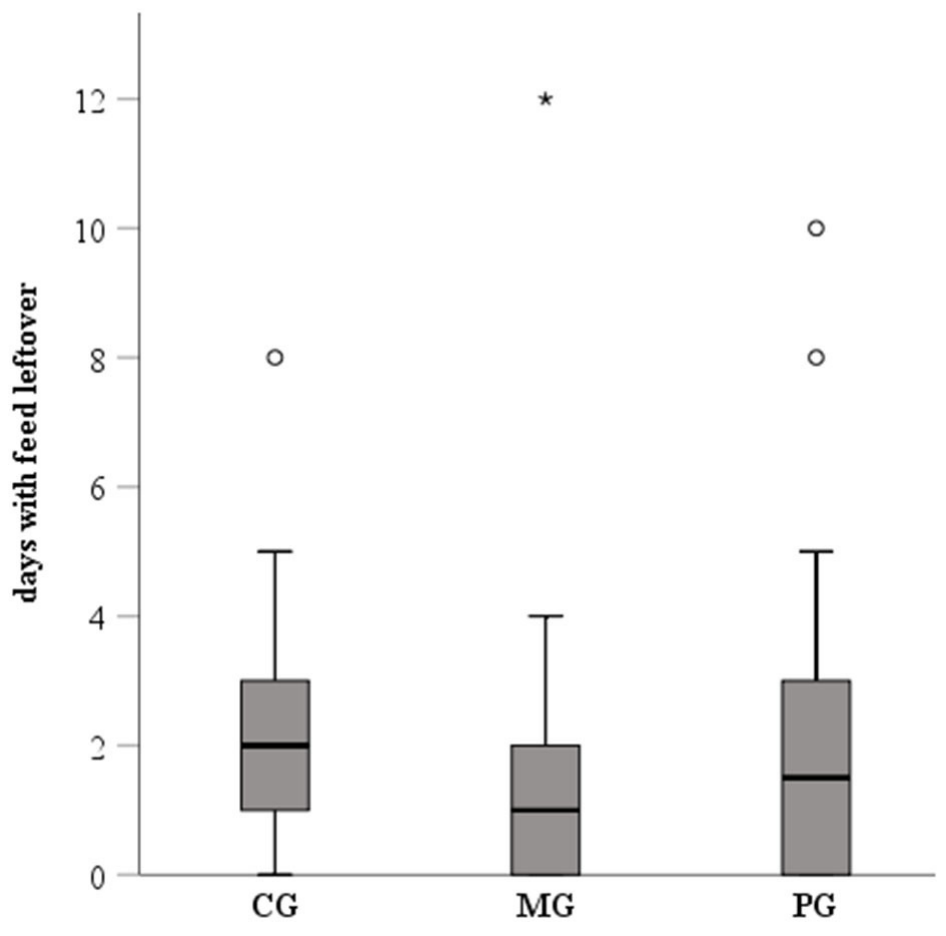
Feed intake (number of days with feed leftover) of sows treated with meloxicam (0.4 mg/kg BW, *n* = 20) or paracetamol (30 mg/kg BW, *n* = 20) for 7 days. Data was recorded from day of entrance to the farrowing unit until 7 days after the last sow had farrowed. Differences between groups were not significant. The * represents an extreme outlier, whereas the ◦ represent outliers.

**Figure 4:**
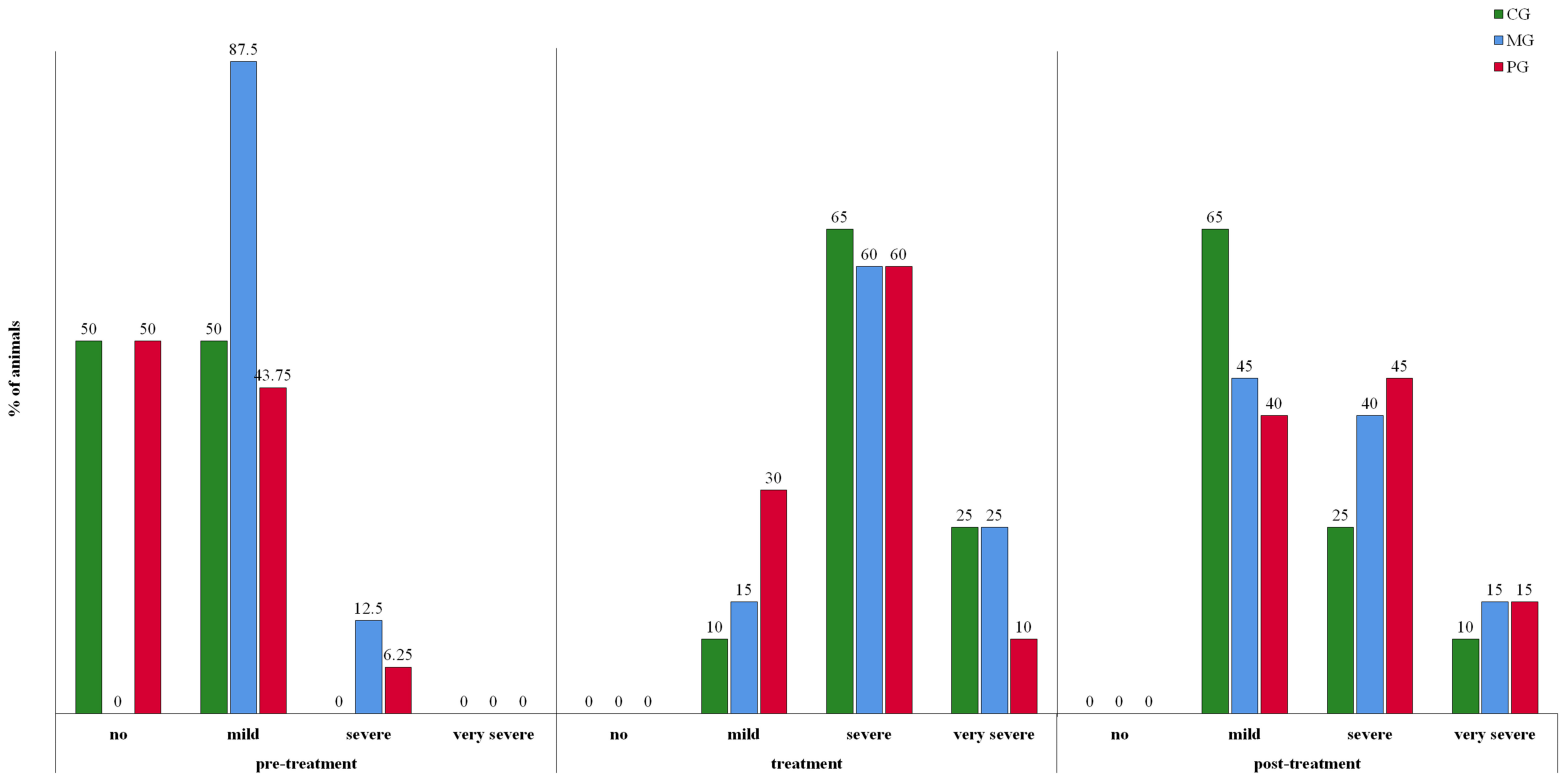
Constipation in sows treated with meloxicam (0.4 mg/kg BW, *n* = 20) or paracetamol (30 mg/kg BW, *n* = 20) for 7 days. Pre-treatment period is defined as the time between day of entrance to the farrowing unit and gestation day 113. Treatment period is defined as the 7 day treatment period from gestation day 113 onwards. Post-treatment period is defined as from day after last treatment until 7 days after the last sow had farrowed. Mild constipation was defined as one or two consecutive days without defecation. If sows did not defecate for 3 or 4 days, it was considered as severe constipation, and if sows did not defecate for five or more days, it was considered as very severe constipation, i.e., obstipation.

Feed leftover was recorded on average on 2.3, 1.7, and 2.3 days in the CG, MG and PG, respectively (Figure 3). Differences between groups were not significant. Considering the whole recording period, 85% (17/20), 65% (13/20), and 70% (14/20) of the sows in the CG, MG and PG, respectively, showed at least once feed leftover. In order to correctly evaluate the degree of constipation, sows with recordings for at least two consecutive days in the different periods were included in the analysis (pre-treatment period: *n* = 47, treatment period: *n* = 60; post-treatment period: *n* = 60). Results are presented in Figure 4. Considering the whole recording period, 35% (7/20), 30% (6/20), and 15% (3/20) of the sows showed obstipation in the CG, MG and PG, respectively. There were no significant differences between the treatment groups in all three periods when taking into account repeated measurements over time (*P* > 0.5).

Retrospective evaluation of the health parameters showed that 45% (9/20), 35% (7/20), and 25% (5/20) of the sows in the CG, MG, and PG, respectively, were defined as clinically PPDS-affected. Due to the limited number of clinically PPDS-affected sows per treatment group, statistical analysis with PPDS-affected and non-affected sows as subjects for all investigated parameters was not possible.

#### Backfat Loss

**Figure 5:**
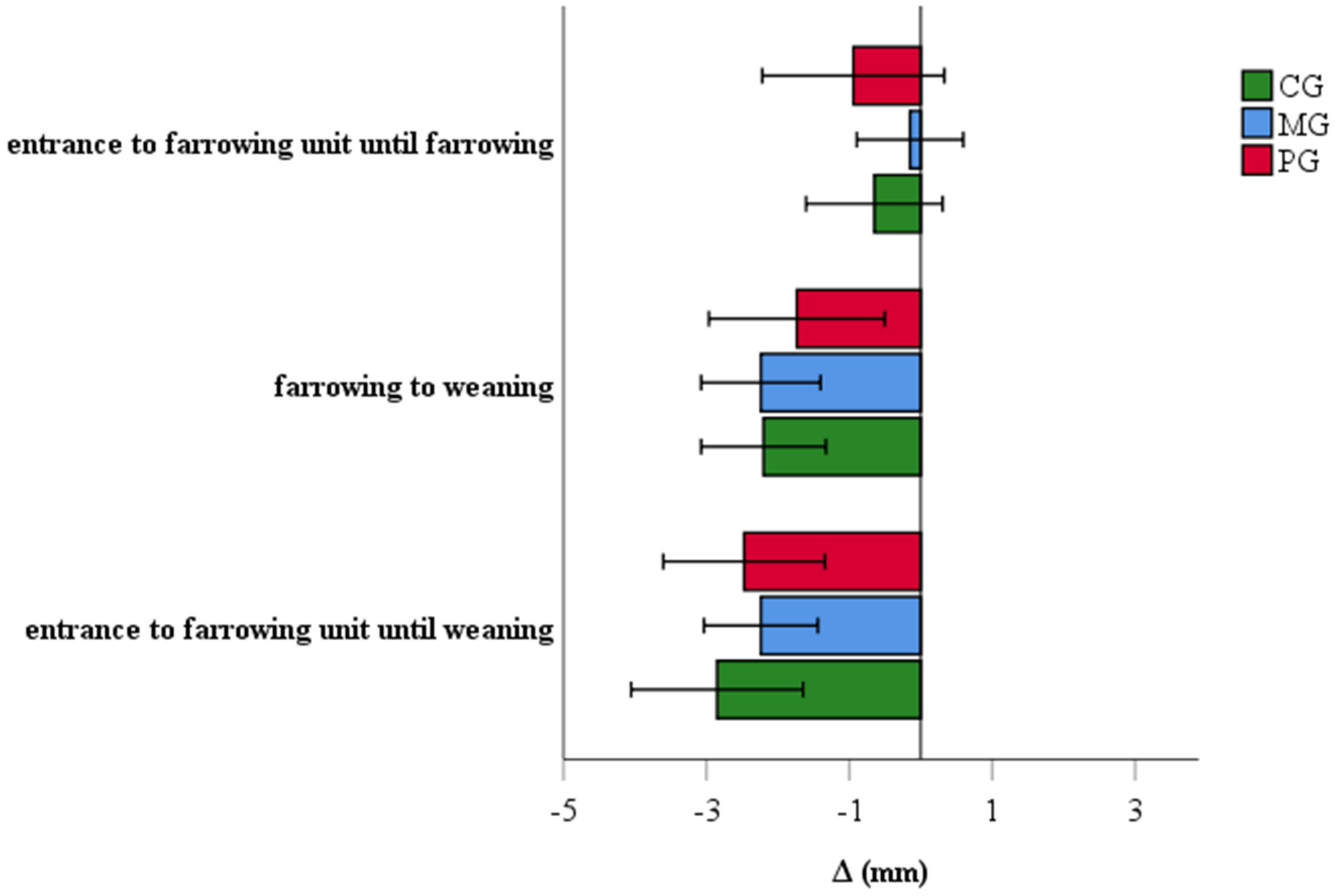
Backfat loss of sows treated with meloxicam (0.4 mg/kg BW, *n* = 20) or paracetamol (30 mg/kg BW, *n* = 20). Differences of backfat loss between groups were not significant.

At the entrance to the farrowing unit, the backfat thickness of sows was 16.7 ± 4.4 mm (mean ± SD), 14.2 ± 3.6 mm and 16.0 ± 4.2 mm in the CG, MG and PG, respectively (*P* = 0.1). At weaning, sows and gilts had an average (± SD) backfat thickness of 13.8 ± 2.9, 11.6 ± 3.2, and 13.1 ± 3.6 mm in the CG, MG and PG, respectively. Backfat at weaning differed significantly between the groups (*P* = 0.02): the MG was associated with a significantly lower backfat at weaning compared to the CG (*P* = 0.02), while non-significant differences were observed for MG-PG (*P* = 0.4) and PG-CG (P > 0.5). Results of backfat loss of the different groups are represented in Figure 5.

#### Systemic Concentrations of Inflammatory Markers

**Figure 6:**
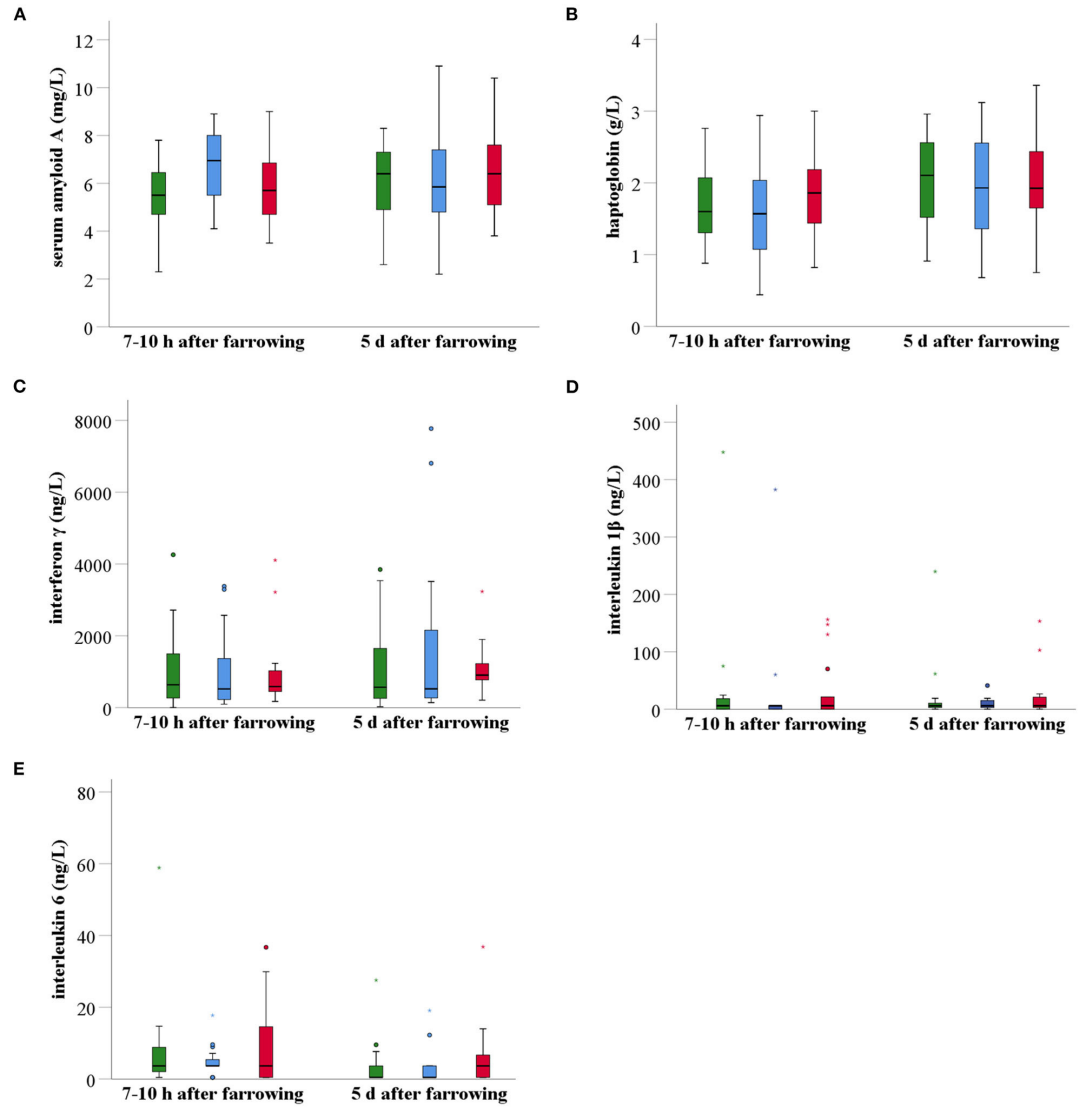
Boxplots of acute phase proteins (SAA, Hp) **(A,B)** and pro-inflammatory cytokines (IFN-γ, IL-1β, IL-6) **(C–E)** in serum of non-treated sows (*n* = 20, green), meloxicam-treated sows (0.4 mg/kg BW, *n* = 20, blue) and paracetamol-treated sows (30 mg/kg BW, *n* = 20, red). Serum samples were taken at two different time points after parturition, i.e., 7–10 h after farrowing and 5 days after farrowing. At 7–10 h postpartum, sows of the MG had a significantly higher mean (± SD) SAA serum concentration than sows of the CG (*P* = 0.028), but not than sows of the PG (*P* > 0.05). No other statistical significant differences were found between groups at any other time point. BW, bodyweight; SAA, serum amyloid A; Hp, haptoglobin; IFN-γ, interferon γ; IL-1β, interleukin 1β; IL-6, interleukin 6.

Shortly after farrowing, sows of the MG had a significantly higher mean (± SD) SAA serum concentration than sows of the CG (*P* = 0.03), but not than sows of the PG (*P* > 0.05). However, at day five after farrowing, no statistically significant differences were observed between groups (P > 0.05). The overall mean (± SD) SAA concentration in serum increased from 5.9 (± 1.6) mg/L shortly after farrowing to 6.2 (± 1.9) mg/L 5 days after farrowing (Figure 6A). Haptoglobin concentrations at both time points were not significantly different between groups (Figure 6B). The overall mean (± SD) serum haptoglobin concentration was 1.7 (± 0.6) g/L at 7–10 h after farrowing and 2.0 (± 0.7) g/L 5 days after farrowing. There were no significant differences between the treatment groups for the serum concentrations of the three investigated pro-inflammatory cytokines, i.e., IFN-γ, IL-1β, and IL-6 (Figures 6C–E). The overall mean (± SD) IFN-γ concentration in serum rose from 964 (± 1,005) ng/L shortly after farrowing to 1,272 (± 1,474) ng/L 5 days after farrowing. In contrast, the overall mean (± SD) IL-1γ concentration in serum decreased from 29.4 (± 79.2) ng/L shortly after farrowing to 16.9 (± 37.8) ng/L 5 days after farrowing. The overall mean (± SD) IL-6 concentration in serum decreased as well, i.e., from 20.0 (± 74.7) ng/L shortly after farrowing to 12.1 (± 47.6) ng/L 5 days after farrowing. The evolution over this 5-day time period in the three treatment groups was not significant.

#### Performance Parameters in Piglets

Colostrum intake, ADWG and the chance of survival were considered as performance parameters in piglets. In total 978 piglets were born from 60 sows that were enrolled in the study: 332 in the CG, 321 in the MG and 325 piglets in the PG. The average birthweight was comparable between groups (Table 3) and in each group approximately one third of the piglets had a birthweight ≤ 1,000 g (109/332, 102/321, and 112/325 piglets in the CG, MG and PG, respectively).

**Table 3:** Effects of meloxicam (0.4 mg/kg BW) and paracetamol (30 mg/kg BW) treatment in sows on their offspring's performance.

	**Control (*n* = 332)**		**Meloxicam (*n* = 321)**		**Paracetamol (*n* = 325)**		
	**Mean**	**SD**	**Mean**	**SD**	**Mean**	**SD**	***P*-value**
BWB (kg)	1.15	0.32	1.15	0.29	1.13	0.30	0.8
BW24 (kg)	1.22	0.36	1.20	0.32	1.21	0.34	0.9
BWW (kg)	4.78	1.51	4.72	1.30	4.76	1.36	0.9
Colostrum intake (g)	301	174	295	138	301	156	0.9
ADWG (g/day)	179	71	182	65	189	69	0.3

*Sows were treated for 7 days from gestation day 113 onwards. BWB, bodyweight at birth; BW24, bodyweight at 24 h; BWW, bodyweight at weaning; ADWG, average daily weight gain*.

**Figure 7:**
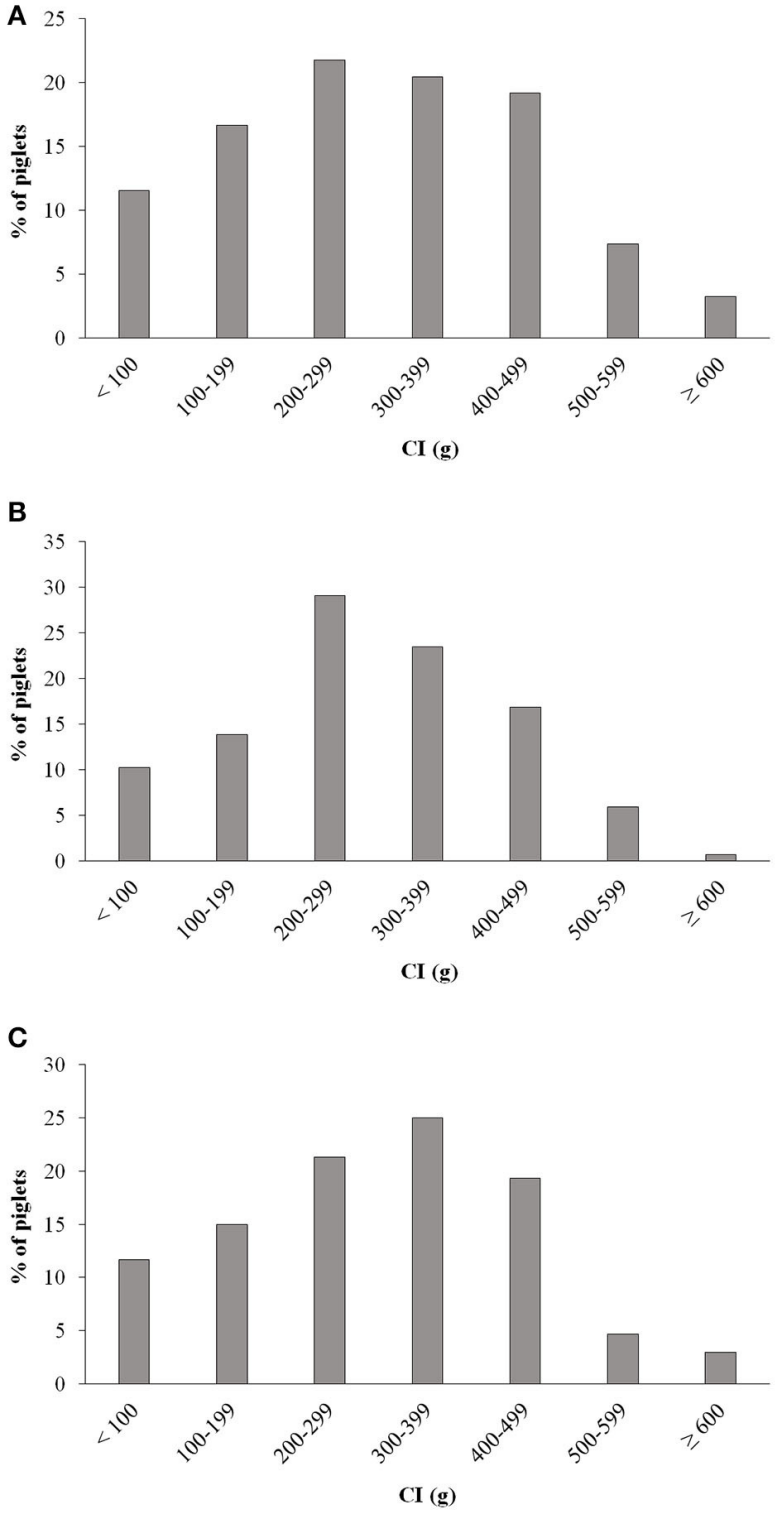
Distribution of colostrum intake (CI) in piglets of **(A)** non-treated sows, **(B)** of sows treated with meloxicam (0.4 mg/kg BW) and **(C)** of sows treated with paracetamol (30 mg/kg BW). BW, bodyweight; CI, colostrum intake.

One, three and four piglets of the CG, MG, and PG, respectively, were missed at the weighing time point at 24 h. Negative colostrum intake was recorded in five, four and five piglets in the CG, MG, and PG, respectively (Table 3). The majority of piglets in all three groups had between 200 and 500 g colostrum intake (Figures 7A–C). However, in each group one fourth or more of the piglets had a colostrum intake lower than 200 g with the highest prevalence in the CG, namely 28.1%.

The survival rate was higher in piglets of treated sows (82.2 and 84.9% in the MG and PG, respectively), than of the non-treated sows (80.1%). Differences between the three groups were not significant. Over the whole lactation period, only five litters were treated for diarrhea: two litters of the CG, two of the MG and one of the PG. Antibiotic treatment could not be evaluated statistically because of the low number of treated litters.

## Discussion

This study investigated whether prophylactic administration of either meloxicam or paracetamol to sows in a farm with a history of PPDS affects their health and performance, as well as the performance of their offspring. Overall, neither meloxicam nor paracetamol administration had an effect on the investigated parameters in this study.

Historically, the sows of the selected farm showed unspecific signs and risk factors for PPDS, such as reduced appetite, constipation and lethargy, and a high preweaning mortality and low weaning weight in piglets. However, there were no records regarding rectal temperature, vaginal discharge or milk production of these hyperprolific Danbred sows with on average 16.8 live born piglets in the last year. Such large litter sizes lead to a longer farrowing duration (in this study 6.9 h on average), which is a major risk of developing PPDS as the risk for postpartum fever and endometritis increases (24–26). Additionally, although sows' CY and piglets' CI are similar to those of other studies with similar litter sizes (27), the ADWG of piglets in the current study was much lower, which might be due to dysgalactia problems during lactation.

In this study, rectal temperature was considered as main efficacy criterion. Five sows per group showed at least once a rectal temperature of ≥ 39.5°C during the whole recording period. Paracetamol-treated sows averaged the lowest with 38.1°C rectal temperature, which was significantly lower than the MG. Our latter observation corroborates the studies of Mainau et al. and Tenbergen et al. (28, 29) reporting no effect of meloxicam treatment with respect to rectal temperature. Furthermore, no effect on rectal temperature after meloxicam administration was detected in a porcine endotoxin-challenge *in vivo* model (30). Claeyé et al. (31) did show a significant reduction (-0.43 °C) of the rectal temperature in sows after administration of the COX inhibitor ketoprofen within 12 h postpartum in sows. Being a non-selective COX inhibitor and acting at both the central and peripheral level, ketoprofen has strong anti-inflammatory effects (32). So far, no research comparing ketoprofen and meloxicam in peripartal sows has been performed. Paracetamol has strong antipyretic effects through its central action. It seems that also in sows with increased rectal temperature, paracetamol has a better effect on lowering rectal temperature than meloxicam, which has mainly peripheral anti-inflammatory and analgesic effects. However, the clinical relevance of the significant difference in this study is not clear, as differences are minimal and the overall rectal temperatures of all three groups are within physiological ranges in sows around parturition.

Mild constipation around farrowing can be physiological due to low fiber content of the feed and restricted feeding (33). However, severe constipation in the peripartal period might cause serious problems, namely prolonged farrowing length and risk of PPDS (34–36). In our study, no significant differences were seen between treatment groups. To date, no studies investigated the effect of paracetamol on those parameters. Previous studies investigated the effect of meloxicam treatment (one administration at farrowing) in healthy sows, but no clear difference was found, although it seemed that sows ate more frequently their first meal of the day when treated (28, 29). A limitation of the current study is thus, that the lack of appetite was only recorded in the morning and therefore might have been underestimated. Viitasaari et al. showed that treatment with another NSAID, ketoprofen, was associated with a significantly shorter duration of constipation and later incidences of feed refusal in peripartal sows, even though constipation could not be completely prohibited (37).

Several inflammatory markers have been suggested to serve as potential biomarkers for identifying PPDS-affected sows (38, 39). Therefore, lower concentrations of APPs and cytokines were expected after treatment with meloxicam or paracetamol in the present study. However, neither meloxicam- nor paracetamol-treated sows had significant lower values in any parameter at any moment in comparison to the untreated sows. Haptoglobin is a major APP described in sows with peripartal disorders. In healthy sows, Hp serum concentrations were 2.09 g/L (range: 0.78–3.84) at 7 ± 2 days postpartum (38), which is in line with the average of 2.0 (± 0.7) g/L at 5 days after farrowing in the current study. Nonetheless, several other studies reported that Hp is significantly increased in PPDS-affected sows in comparison to healthy sows (40–42). Regarding both APPs, Viitasaari et al. (37) showed that SAA varies in the peripartal period in non-treated vs. ketoprofen-treated healthy sows. More specifically, sows treated with ketoprofen during 3 days had systematically higher SAA concentrations in their serum than the placebo group. However, in apparent contradiction, no treatment effect was noticed regarding Hp concentrations. Therefore, authors attributed the increased SAA concentrations merely to a transient tissue irritation after ketoprofen injection. This and our data are also in line with a study of artificially induced clinical PPDS signs, where sows were challenged with two different doses of *Escherichia coli* endotoxin, and treatment of meloxicam did not lead to any significant reduction of APPs. Nevertheless, substantial inter-animal fluctuations of APPs in plasma within each group were seen, as it was also the case in this study (30). Increased body temperature is one of the cardinal signs of an inflammatory process. However, in the present study less than half of the sows had rectal temperatures ≥ 39.5°C. The lack of clinical signs of inflammation might explain why no notifiable differences in APPs and cytokines were found between groups. Overall it can be concluded that administration of meloxicam and paracetamol for 7 days did not reduce the circulating levels of both type of investigated inflammatory markers in the sows.

Postpartum dysgalactia syndrome leads to starvation of the offspring, growth retardation and a high preweaning mortality, whereof most of the piglets die within the 1st week of life (41, 43). Increasing litter sizes and lower birth weights in the hyperprolific sows cause additional risk factors for an elevated mortality during lactation (44). In our study, the average birthweight was rather low (1.1 ± 0.3 kg) in comparison to other studies where birthweights averaged over 1.4 kg (27, 29, 31, 45). The total born piglets per litter in this farm was high (20.6 ± 4.6 piglets), which might explain these lower birthweights. The ADWG was low compared to another study in healthy sows and with similar numbers of live born piglets (236.1 ± 102 g) (27). This can be a sign of dysgalactia problems in the sows of the present study. Although no significant differences were found in all these parameters between the three groups, the ADWG was higher in the piglets of treated sows (+3 g and +10 g in the MG and PG, respectively). Tenbergen et al. (29) could not identify a significant increase in ADWG after intramuscular meloxicam administration, while Mainau et al. (45) described a significant higher ADWG (+19 g) after oral meloxicam treatment. Two studies investigating ADWG of piglets of sows treated with another NSAID, i.e., ketoprofen, also did not show a significant improvement of ADWG after ketoprofen treatment (31, 37).

In the present study, the overall preweaning mortality averaged 23.4%, with the highest percentages present in piglets of non-treated sows. Piglet mortality within the first 24 h was rather low (5.5%), which can be explained by the strict and permanent supervision. However, the first-week mortality was high (19.5%). The precise reasons of death were not recorded for each pig, but according to literature, 1st-week mortality is mainly due to crushing, starvation and low birth weight (44, 46). No significant differences were recorded in this study after preventive use of either meloxicam or paracetamol, which is in overall agreement with the literature regarding meloxicam treatment in sows (28, 29, 45). Some studies using another NSAID, ketoprofen, within 12 h postpartum in PPDS-affected sows showed a significant overall lower preweaning mortality within the 1st week of life (47, 48). Conversely, Claeyé et al. (31) only detected a trend for increased survival of piglets in ketoprofen-treated sows suffering from PPDS. Differences between outcomes of studies might be mainly due to different study designs, especially differences in presence of evident clinical signs in the study population, or to different treatment strategies.

Literature about oral administration of meloxicam and paracetamol in sows is scarce. In order to verify a correct administration of the respective drug, a single plasma sample was taken at the presumed T_max_ 1 day after farrowing. The mean plasma concentration of meloxicam averaged 786 ng/mL, which is similar than in the pharmacokinetic study of Pairis-Garcia et al. (20) (0.5 mg/kg BW meloxicam, once per os), namely 1,070 ng/mL. The variability was also in the same range, i.e., 320–1,949 ng/mL compared to 645–1,749 ng/mL. Sows of the PG had a mean plasma concentration of 3,548 ng/mL with a range from 281 to 41,738 ng/mL. Neirinckx et al. (21) observed a mean (± SD) paracetamol (10 mg/kg BW paracetamol, once per os) plasma concentration of 4,410 (± 2,240) ng/mL, which is higher than in the present study, albeit still within the same range. One outlier with a plasma concentration of 41,738 ng/mL was measured in the PG. This sow was the oldest one in the group (9th parity), and she received five out of seven treatments at moment of blood sampling. Looking at the meloxicam-treated sows, also here the highest concentration (1,949 ng/mL) was observed in the oldest sow (13th parity), which had received six out of seven treatments at moment of blood sampling. The number of animals per parity and per amount of treatments was too small in this study to investigate an effect of parity and number of treatments on the drug plasma concentration. Overall, drug plasma concentrations suggest that an appropriate oral dose was administered to the peripartal sows.

In pigs, several studies have been conducted to investigate the influence of NSAIDs, namely using the COX-1 and COX-2 inhibitor indomethacin. In 1981, Nara and First (49) described a prolonged gestation length in sows up to 120 days (vs. 114.9 days in the control group), as well as delayed prepartum hormonal changes, a lower percentage of live born piglets as well as a delayed luteolysis after administration of indomethacin from gestation day 109 until day 116. An earlier study showed also the inhibition of proper luteolysis and thus absence of decline in progesterone levels, as well as lower weight gains of piglets after administration of indomethacin over a 10–14-days period (50). A more recent study described no prolonged gestation length, signs of toxicity nor differences in time to delivery in gilts after indomethacin administration (4 mg/kg BW, 2 h after start of prepartum nest building behavior) (51). Hence the time point of administration and the duration seems to play a major role of the effect of NSAIDs. So far, no studies investigated the potential effect of meloxicam or paracetamol in pregnant sows, however both products used in the current study are registered to be given during gestation and lactation of sows. Hirsch et al. investigated different reproduction parameters upon meloxicam treatment (1.5 mg/kg BW over 3 days) in cattle. No differences in pregnancy rate, gestation or parturition length were observed (52). In the present study, a significant longer gestation length was seen in the PG (116.3 days vs. 115.3 and 115.9 days in the CG and MG, respectively). However, this observation needs to be interpreted with caution, as it might have been caused artificially. Investigators were blinded to treatment and sows were enrolled upon onset of farrowing. If the birth of the first piglet was missed, the sow was not enrolled in the study. Retrospectively it turned out that the two sows that were missed in total, belonged to the PG. Therefore, a prolonged gestation length after paracetamol treatment cannot be unequivocally stated based on the present study. Additionally, historical farm records show an average gestation length of 116 days in this farm, thus the gestation length of the PG was still in line with earlier records.

The retrospective evaluation of the sows revealed only a limited number of sows being categorized as clinically PPDS-affected. Yet, this should not be viewed as a big limitation of the study since PPDS can often be subclinical, as is the case in this study, which causes also economic losses due to high pre-weaning mortality and low ADWG in the piglets. The aim of this study was to investigate the prophylactic effect of meloxicam and paracetamol in sows of a farm with a history of PPDS, either with or without clinical signs in the sows. Therefore, it was a challenge to investigate, whether it is relevant to administer a NSAID prior to farrowing in those visually healthy sows as well as in some sows presenting mild clinical signs. Nonetheless, the insufficient presence of clinical signs complicates the evaluation of a therapeutic effect of the administered drugs. It cannot be ruled out, that missing significant differences between groups can be attributed to this. Another limitation of this study is that no information is available on possible side-effects of the drugs as culled sows were not further examined in the slaughterhouse. In fatteners, long time (62 days) oral administration of meloxicam did not cause any side effects based on clinical and pathological examination (53). As we however cannot translate those results to our study (different age group, other circumstances), necropsies of culled sows should be included in future studies to confirm this. Finally, the question of profitability of prophylactic treatment of a herd remains open if no significant differences are observed.

## Conclusion

This study is the first one according to the authors' knowledge investigating preventive oral administration of meloxicam or paracetamol over 7 days to sows suffering from PPDS. Neither meloxicam nor paracetamol ameliorated the PPDS situation of the sows in this farm, where sows were mainly non-symptomatic. Additionally, no previous study has investigated the effect of COX inhibition shortly before farrowing on reproductive performance parameters in sows so far. Overall, there was no evidence of disadvantageous effects on the sows related to treatment with either of both COX inhibitors. Nonetheless, future studies are warranted which should include more batches, especially with more sows showing clinical signs of PPDS. Further research is also warranted to assess the effect of NSAID treatment duration and onset of this anti-inflammatory treatment.

## Data Availability Statement

The raw data supporting the conclusions of this article will be made available by the authors, without undue reservation.

## Ethics Statement

The animal study was reviewed and approved by Ethical Committee of the Faculty of Veterinary Medicine and the Faculty of Bioscience Engineering, Ghent University. Written informed consent was obtained from the owners for the participation of their animals in this study.

## Author Contributions

AS contributed to study design, sample collection, sample analysis, and wrote the manuscript. IC performed the statistical data analysis and wrote sections of the manuscript. JV contributed to sample collection. EB, EM, and AC contributed to editing manuscript. MD and DM contributed to study design, data analysis, and manuscript editing. All authors contributed to manuscript revision, read, and approved the submitted version.

## Conflict of Interest

The authors declare that the research was conducted in the absence of any commercial or financial relationships that could be construed as a potential conflict of interest.

## Abbreviations

ADWG: average daily weight gain
BW: bodyweight
BW24: bodyweight 24 h after birth
BWB: bodyweight at birth
BWW: bodyweight at weaning
CG: non-treated control group
CI: colostrum intake
COX: cyclooxygenase
CY: colostrum yield
Hp: haptoglobin
HPLC: high performance liquid chromatography
IFN-γ: interferon γ
IgG: immunoglobulin G
IL-1β: interleukin 1β
IL-6: interleukin 6
LOD: limit of detection
LOQ: limit of quantification
MG: meloxicam-treated group
NSAID: non-steroidal anti-inflammatory drug
PG: paracetamol-treated group
PGF2α: prostaglandin F2α
PPDS: postpartum dysgalactia syndrome
SAA: serum amyloid A
TNF-α: Tumor necrosis factor α
WG: weight gain.
