# Supplementary material for: Prophylactic Use of Meloxicam and Paracetamol in Peripartal Sows Suffering From Postpartum Dysgalactia Syndrome
Source: Front Vet Sci. 2020 Dec 8;7:603719. doi: 10.3389/fvets.2020.603719 (PMC7793981; doi:10.3389/fvets.2020.603719)
Supplement: Supplementary file 1 [file Data_Sheet_1.PDF]

Supplementary Material

Supplementary file 1: SPC of Metacam 15 mg/ml oral suspension for pigs

https://www.ema.europa.eu/en/documents/product-information/metacam-epar-product-information_en.pdf

1. NAME OF THE VETERINARY MEDICINAL PRODUCT

Metacam 15 mg/ml oral suspension for pigs

2. QUALITATIVE AND QUANTITATIVE COMPOSITION

One ml contains:

Active substance(s):

Meloxicam	15 mg

Excipient(s):

Sodium benzoate	1.5 mg

For a full list of excipients, see section 6.1.

3. PHARMACEUTICAL FORM

Oral suspension
Yellowish viscous oral suspension with a green tinge.

4. CLINICAL PARTICULARS

4.1 Target species

Pigs

4.2 Indications for use, specifying the target species

For use in non-infectious locomotor disorders to reduce the symptoms of lameness and inflammation. For adjunctive therapy in the treatment of puerperal septicaemia and toxaemia (Mastitis-Metritis- Agalactia syndrome MMA) with appropriate antibiotic therapy.

4.3 Contraindications

Do not use in pigs suffering from impaired hepatic, cardiac or renal function and haemorrhagic disorders, or where there is evidence of ulcerogenic gastrointestinal lesions.
Do not use in case of hypersensitivity to the active substance or to any of the excipients.

4.4 Special warnings

None.

4.5 Special precautions for use

Special precautions for use in animals
If adverse reactions occur, treatment should be discontinued and the advice of a veterinarian should be sought.
Avoid use in very severely dehydrated, hypovolaemic or hypotensive pigs which require parenteral rehydration, as there may be a potential risk of renal toxicity.
Special precautions to be taken by the person administering the veterinary medicinal product to animals
People with known hypersensitivity to Non-Steroidal Anti-Inflammatory Drugs (NSAIDs) should avoid contact with the veterinary medicinal product.
In case of accidental ingestion, seek medical advice immediately and show the package leaflet or the label to the physician.

4.6 Adverse reactions (frequency and seriousness)

None.

4.7 Use during pregnancy, lactation or lay

Can be used during pregnancy and lactation.

4.8 Interaction with other medicinal products and other forms of interaction

Do not administer concurrently with glucocorticosteroids, other non-steroidal anti-inflammatory drugs or with anticoagulant agents.

4.9 Amounts to be administered and administration route

Oral suspension to be administered at a dosage of 0.4 mg/kg body weight (i.e. 2.7 ml/100 kg) in combination with antibiotic therapy, as appropriate. If required, a second administration of Meloxicam can be given after 24 hours.
In cases of MMA with severely disturbed general demeanour (e.g. anorexia) the use of Metacam 20 mg/ml solution for injection is recommended.

To be administered preferably mixed with a small quantity of feed. Alternatively to be given prior to feeding, or directly into the mouth.
The suspension should be given using the measuring syringe provided in the package. The syringe fits onto the bottle and has a kg-body weight scale.

Shake well before use.

After administration of the veterinary medicinal product, close the bottle by replacing the cap, wash the measuring syringe with warm water and let it dry.

4.10 Overdose (symptoms, emergency procedures, antidotes), if necessary

In case of overdose symptomatic treatment should be initiated.

4.11 Withdrawal period

Meat and offal: 5 days.

5. PHARMACOLOGICAL PROPERTIES

Pharmacotherapeutic group: Antiinflammatory and antirheumatic products, non-steroids (oxicams). ATCvet code: QM01AC06.

5.1 Pharmacodynamic properties

Meloxicam is a Non-Steroidal Anti-Inflammatory drug (NSAID) of the oxicam class which acts by inhibition of prostaglandin synthesis, thereby exerting anti-inflammatory, analgesic, anti-exudative and antipyretic effects. It reduces leukocyte infiltration into the inflamed tissue. To a minor extent it also inhibits collagen-induced thrombocyte aggregation. Meloxicam also has anti-endotoxic properties because it has been shown to inhibit production of thromboxane B_2 induced by intravenous *E. coli* endotoxin administration in pigs.

5.2 Pharmacokinetic particulars

Absorption
After a single oral dose of 0.4 mg meloxicam/kg a C_{max} value of 0.81 µg/ml was reached after 2 hours.

Distribution
More than 98 % of meloxicam is bound to plasma proteins. The highest meloxicam concentrations are to be found in liver and kidney. Comparatively low concentrations are detectable in skeletal muscle and fat.

Metabolism
Meloxicam is predominantly found in plasma. Bile and urine contain only traces of the parent compound. Meloxicam is metabolised to an alcohol, an acid derivative and to several polar metabolites. All major metabolites have been shown to be pharmacologically inactive.

Elimination
After oral administration the mean plasma elimination half-life is approximately 2.3 hours. Approximately 50 % of the administered dose is eliminated via urine and the remainder via faeces.

6. PHARMACEUTICAL PARTICULARS

6.1 List of excipients

Sodium benzoate
Sorbitol, liquid
Glycerol
Saccharin sodium
Xylitol
Sodium dihydrogen phosphate
dihydrate Silica, colloidal anhydrous
Hydroxyethylcellulose
Citric acid
Honey aroma
Water, purified

6.2 Incompatibilities

None known.

6.3 Shelf life

Shelf-life of the veterinary medicinal product as packaged for sale: 3 years. Shelf-life after first opening of the immediate packaging: 6 months.

6.4 Special precautions for storage

This veterinary medicinal product does not require any special storage conditions.

6.5 Nature and composition of immediate packaging

Cardboard box containing one polyethylene bottle of 100 ml or 250 ml with a polyethylene tip adapter, a tamper proof child resistant closure and a measuring syringe. Not all pack sizes may be marketed. Special precautions for the disposal of unused veterinary medicinal product or waste materials derived from the use of such products

Any unused veterinary medicinal product or waste materials derived from such veterinary medicinal products should be disposed of in accordance with local requirements.

7. MARKETING AUTHORISATION HOLDER

Boehringer Ingelheim Vetmedica
GmbH 55216 Ingelheim/Rhein
GERMANY

8. MARKETING AUTHORISATION NUMBERS

EU/2/97/004/041 100 ml
EU/2/97/004/042 250 ml

9. DATE OF FIRST AUTHORISATION/RENEWAL OF THE AUTHORISATION

Date of first authorisation: 07.01.1998
Date of last renewal: 06.12.2007

10. DATE OF REVISION OF THE TEXT

Detailed information on this veterinary medicinal product is available on the website of the European Medicines Agency http://www.ema.europa.eu/.

11. PROHIBITION OF SALE, SUPPLY AND/OR USE

Not applicable.

Supplementary file 2: SPC of Pracetam 400 mg/ml solution for use in drinking water for pigs

https://myhealthbox.eu/en/view/1980299/ef274c34eae74c35857ab485f273345b/leaflet

1. NAME OF THE VETERINARY MEDICINAL PRODUCT

Pracetam 400 mg/ml solution for use in drinking water for pigs
Pracetam Vet 400 mg/ml solution for use in drinking water for pigs (DK)
Pracetam 40% solution buvable pour porc (FR)

2. QUALITATIVE AND QUANTITATIVE COMPOSITION

Each ml contains:

Active substance:

Paracetamol ....400 mg

Excipients:

For the full list of excipients, see section 6.1.

3. PHARMACEUTICAL FORM

Solution for use in drinking water. Clear viscous pink solution.

4. CLINICAL PARTICULARS

4.1 Target species

Pigs

4.2 Indications for use, specifying the target species

In pigs:

Symptomatic treatment of fever in the context of respiratory diseases in combination with an appropriate anti-infective therapy, if necessary.

4.3 Contraindications

- Do not use in animals with known hypersensitivity to paracetamol and to any other ingredients of the product
- Do not use in animals with severe hepatic impairment
- Do not use in animals with severe renal impairment. See also section 4.8
- Do not use in animals suffering from dehydration or hypovolemia

4.4 Special warnings for each target species

Animals with reduced water intake and/or disturbed general condition have to be treated parenterally. In case of combined viral and bacterial etiology of the disease, an appropriate anti-infective therapy should be given concomitantly.

4.5 Special precautions for use

Special precautions for use in animals
The anti-pyretic effect of the product is expected at 12 - 24 hours after the onset of treatment.

Special precautions to be taken by the person administering the veterinary medicinal product to animals
Do not handle the product if you know you are hypersensitive to paracetamol.
Wear appropriate protective clothing, gloves, goggles and mask when handling the product.
To rule out any risk of ingestion it is recommended not to eat or drink while using the product and to wash hands after use.

If the product comes in contact with the skin or eyes, rinse immediately with a large amount of water. If symptoms persist, seek medical advice.
In the case of accidental ingestion, seek medical advice.

4.6 Adverse reactions (frequency and seriousness)

In rare cases, at therapeutic doses, transient soft feces can occur and can persist for up to 8 days after the withdrawal of treatment. This does not have any effect on the general condition of animals, and resolves without any specific treatment.

4.7 Use during pregnancy, lactation or lay

Studies in laboratory animals have not detected any teratogenic or foetotoxic effects at therapeutic doses. The administration of the product up to three times the recommended dose, during pregnancy or lactation, did not result in adverse effects. As such the product may be administered during pregnancy and lactation.

4.8 Interaction with other medicinal products and other forms of interaction

Concurrent administration of nephrotoxic drugs should be avoided.

4.9 Amounts to be administered and administration route

In drinking water use
30 mg of paracetamol per kg body weight per day, for 5 days, orally, administered in the drinking water, equivalent to 0.75 ml of oral solution per 10 kg body weight per day for 5 days.

The intake of medicated drinking water depends on the clinical condition of the animals. In order to obtain a correct dosage, the concentration in the drinking water must be adjusted accordingly.

Recommendation for dissolution:
First add, the necessary quantity of water for the preparation of the final solution in the container.

Then add the product while stirring the solution.
Preferably prepare, the solution in water at ambient temperature (20°C – 25°C).
For water at 25°C, there is an upper concentration limit of 40ml of product per liter of drinking solution.

When using the product with a water proportioner, adjust the setting to 3% - 5%. Do not set proportioners below 3%.

The solution should be prepared freshly every 24 hours. No other source of drinking water should be available during the medication period.

4.10 Overdose (symptoms, emergency procedures, antidotes), if necessary

After administration of 5 times the recommended dose of paracetamol, liquid faeces with solid particles may occasionally occur. It does not have any effect on general body condition of animals. Acetylcysteine can be used in case of accidental overdose.

4.11 Withdrawal period(s)

Meat and offal: zero days.

5. PHARMACOLOGICAL PROPERTIES

Pharmacotherapeutic group: Other Analgesics and Antipyretics ATCvet code: QN02BE01

5.1 Pharmacodynamic properties

Paracetamol or acetaminophen or N-acetyl-p-aminophenol is a paraminophenol derivative with analgesic and antipyretic properties.

5.2 Pharmacokinetic particulars

Absorption: Paracetamol is rapidly and almost completely absorbed after oral administration (bioavailability of about 90% after administration in the drinking water). Peak concentrations are reached in a little less than 2 hours after ingestion.

Metabolism: Paracetamol is mainly metabolised in the liver. The two major metabolic pathways are conjugation to glucuronate and conjugation to sulphate. The latter route is rapidly saturable at dosages higher than therapeutic doses. A minor pathway, catalysed by cytochrome P450 (CYP), leads to the formation of the intermediary reagent, N-acetyl-benzoquinoneimine which, under normal conditions of use, is rapidly detoxified by reduced glutathione and removed in urine after conjugation with cystein and mercapturic acid. On the contrary, after massive intoxication, the quantity of this toxic metabolite is increased.

Elimination: Paracetamol is mainly eliminated in the urine. In the pig, 63% of the ingested dose is eliminated by the kidneys in 24 hours mainly conjugated to glucuronate and suphate. Less than 5% is eliminated in unchanged form. The elimination half-life is approximately 5 hours.

6. PHARMACEUTICAL PARTICULARS

6.1 List of excipients

Dimethyl sulfoxide
Ponceau 4R (E124)
Macrogol 300

6.2 Incompatibilities

The product has been proved to be physically-chemically compatible with the actives substances Amoxicillin, Sulfadiazine/Trimethoprime, Doxycycline, Tylosine, Tetracycline, Colistin.

In the absence of compatibility studies, this veterinary medicinal product must not be mixed with other veterinary medicinal products.

6.3 Shelf life

Shelf life of the veterinary medicinal product as packaged for sale: 3 years.
Shelf life after first opening the immediate packaging: 3 months
Shelf life after dilution to directions: 24 hours

6.4 Special precautions for storage

This veterinary medicinal product does not require any special storage conditions.

6.5 Nature and composition of immediate packaging

- High density polyethylene bottle
- High density polyethylene screwcap
- Polyethylene – Polyethylene – Polyethylene – seal (500 ml bottle)
- Polyethylene-aluminium-wax-paper-low density polyethylene seal (1-l bottle)
- Polyethylene-PET-aluminium-wax-cardboard seal (2.5-l and 5-l bottles)
- Polypropylene screwcap (1-l and 5-l bottle)
- Silicone seal (for polypropylene screwcap of 1-l and 5-l bottle)

Not all pack sizes may be marketed.

6.6 Special precautions for the disposal of unused veterinary medicinal product or waste materials derived from the use of such products

Any unused veterinary medicinal product or waste materials derived from such veterinary medicinal product should be disposed of in accordance with local requirements.

7. MARKETING AUTHORISATION HOLDER

Ceva Animal Health Ltd Unit 3,
Anglo Office Park White Lion
Road Amersham
Buckinghamshire
HP7 9FB

8. MARKETING AUTHORISATION NUMBER

Vm 15052/4130

9. DATE OF FIRST AUTHORISATION

06 September 2016

10. DATE OF REVISION OF THE TEXT

May 2017

Supplementary table 1. Feed composition of gestation, transition and lactation feed.

	Gestation	Transition	Lactation
**Water (%)**	10	9.68	8.05
**Dry matter (%)**	90	90.32	91.95
**Crude ash (%)**	5.04	5.9	6.4
**Crude protein (%)**	10.52	13.96	13.81
**Crude fat (%)**	3.31	6.52	5.9
**Crude fiber (%)**	6.09	5.25	6.29
**Other carbohydrates (%)**	65.05	58.66	59.55
**ADF (%)**	12.54	11.42	12.23
**NDF (%)**	17.21	18.23	16.7
**Boron (mg/kg)**	7.73	12	9.39
**Calcium (mg/kg)**	5340	7690	14900
**Copper (mg/kg)**	14	31.2	32.7
**Iron (mg/kg)**	261	228	347
**Potassium (mg/kg)**	6990	8340	8640
**Magnesium (mg/kg)**	1790	2770	2580
**Manganese (mg/kg)**	81.8	98.4	116
**Sodium (mg/kg)**	1640	1850	2240
**Phosphor (mg/kg)**	4280	4990	6940
**Zinc (mg/kg)**	99.3	125	141

Abbreviations: ADF, acid detergent fiber; NDF, neutral detergent fiber

Supplementary table 2. Meloxicam (0.4 mg/kg BW) and paracetamol (30 mg/kg BW) concentrations in plasma of sows at T_{max} one day after farrowing. T_{max} was defined at 30 minutes and 2.5 hours post treatment for paracetamol and meloxicam, respectively.

	**Meloxicam (n=20)**				**Paracetamol (n=20)**		
	**Parity**	**Treatment**	**ng/mL**		**Parity**	**Treatment**	**ng/mL**
Sow 1	2	3	671.2	Sow 1	1	3	802.4
Sow 2	2	3	430.1	Sow 2	4	4	1097.2
Sow 3	3	4	802.8	Sow 3	6	4	3688.1
Sow 4	3	4	943.3	Sow 4	3	4	446.2
Sow 5	2	4	672.8	Sow 5	3	4	3520.1
Sow 6	2	4	650.7	Sow 6	4	4	5223.2
Sow 7	2	4	469.7	Sow 7	5	4	1168.9
Sow 8	2	4	479.7	Sow 8	9	5	41738.4
Sow 9	1	4	320.2	Sow 9	3	5	974.3
Sow 10	3	4	1213.1	Sow 10	6	5	280.8
Sow 11	5	5	1010.6	Sow 11	2	5	373.5
Sow 12	5	5	715.4	Sow 12	1	5	495.7
Sow 13	1	5	385	Sow 13	4	6	4648.2
Sow 14	1	5	452.3	Sow 14	6	6	629.5
Sow 15	1	5	490	Sow 15	5	6	3212.1
Sow 16	13	6	1948.8	Sow 16	2	6	350.3
Sow 17	3	6	839.5	Sow 17	1	6	359.7
Sow 18	4	6	1618.8	Sow 18	1	6	846.1
Sow 19	2	6	937.4	Sow 19	2	7	378.5
Sow 20	5	6	660	Sow 20	4	7	718

Treatment, number of treatments received at blood sampling; T_{max} , time point of maximal concentration of a drug in plasma
